# Acceptance, Satisfaction, and Preference With Telemedicine During the COVID-19 Pandemic in 2021-2022: Survey Among Patients With Chronic Pain

**DOI:** 10.2196/53154

**Published:** 2024-04-29

**Authors:** Michael Alexander Harnik, Alina Scheidegger, Larissa Blättler, Zdenek Nemecek, Thomas C Sauter, Andreas Limacher, Florian Reisig, Martin grosse Holtforth, Konrad Streitberger

**Affiliations:** 1 Department of Anaesthesiology and Pain Medicine Inselspital, Bern University Hospital University of Bern Bern Switzerland; 2 University Hospital Würzburg, Department of Anaesthesiology, Intensive Care, Emergency and Pain Medicine, Centre for Interdisciplinary Pain Medicine Würzburg Germany; 3 Psychosomatic Medicine, Department of Neurology Inselspital, Bern University Hospital University of Bern Bern Switzerland; 4 Department of Emergency Medicine Inselspital, Bern University Hospital University of Bern Bern Switzerland; 5 Emergency Telemedicine University of Bern Bern Switzerland; 6 Department of Clinical Research University of Bern Bern Switzerland; 7 Clinical Psychology and Psychotherapy Department of Psychology University of Bern Bern Switzerland

**Keywords:** acceptance, satisfaction, patient preferences, COVID-19 pandemic, health care providers, phone consultations, pain therapy, eHealth services, patient care, health care delivery, telemedicine, chronic pain, preference

## Abstract

**Background:**

The COVID-19 pandemic has forced many health care providers to make changes in their treatment, with telemedicine being expanded on a large scale. An earlier study investigated the acceptance of telephone calls but did not record satisfaction with treatment or patients’ preferences. This warranted a follow-up study to investigate acceptance, satisfaction, and preferences regarding telemedicine, comprising of phone consultations, among health care recipients.

**Objective:**

The primary aim was to assess the acceptance and satisfaction of telemedicine during the subsequent months of 2021-2022, after the initial wave of the COVID-19 pandemic in Switzerland. Furthermore, we aimed to assess patients’ preferences and whether these differed in patients who had already experienced telemedicine in the past, as well as correlations between acceptance and satisfaction, pain intensity, general condition, perception of telemedicine, and catastrophizing. Finally, we aimed to investigate whether more governmental restrictions were correlated with higher acceptance.

**Methods:**

An anonymous cross-sectional web-based survey was conducted between January 27, 2021, and February 4, 2022, enrolling patients undergoing outpatient pain therapy in a tertiary university clinic. We conducted a descriptive analysis of acceptance and satisfaction with telemedicine and investigated patients’ preferences. Further, we conducted a descriptive and correlational analysis of the COVID-19 stringency index. Spearman correlation analysis and a chi-square test for categorical data were used with Cramer *V* statistic to assess effect sizes.

**Results:**

Our survey was completed by 60 patients. Telemedicine acceptance and satisfaction were high, with an average score of 7.6 (SD 3.3; on an 11-point Numeric Rating Scale from 0=not at all to 10=completely), and 8.8 (SD 1.8), respectively. Respondents generally preferred on-site consultations to telemedicine (n=35, 58% vs n=24, 40%). A subgroup analysis revealed that respondents who already had received phone consultation, showed a higher preference for telemedicine (n/N=21/42, 50% vs n/N=3/18, 17%; *χ*^2^_2_ [N=60]=7.5, *P*=.02, Cramer *V*=0.354), as well as those who had been treated for more than 3 months (n/N=17/31, 55% vs n/N=7/29, 24%; *χ*^2^_2_ [N=60]=6.5, *P*=.04, Cramer *V*=0.329). Acceptance of telemedicine showed a moderate positive correlation with satisfaction (*r*_s_{58}=0.41, *P*<.05), but there were no correlations between the COVID-19 stringency index and the other variables.

**Conclusions:**

Despite high acceptance of and satisfaction with telemedicine, patients preferred on-site consultations. Preference for telemedicine was markedly higher in patients who had already received phone consultations or had been treated for longer than 3 months. This highlights the need to convey knowledge of eHealth services to patients and the value of building meaningful relationships with patients at the beginning of treatment. During the COVID-19 pandemic, the modality of patient care should be discussed individually.

## Introduction

Throughout the COVID-19 pandemic, there have been various measures to curb the spread of the virus, including restrictions on nonurgent care. In Switzerland, this resulted in the temporary suspension of most outpatient visits and the switch to telemedicine, that is, mostly phone consultations. This trend has been encouraged by various interest groups and associations advocating the rapid transition to remote services [1], especially because it can be argued that the COVID-19 pandemic not only exacerbates already persistent pain conditions but also increases the incidence of new-onset pain [2]. While some studies have examined the efficacy of eHealth for the treatment of several conditions (eg, osteoarthritis [3], musculoskeletal conditions [4,5], and chronic pain [6]), evidence from the patients’ perspective remains scarce, so that patients’ preferences are largely unknown, especially during this new era of the COVID-19 pandemic.

A previous survey conducted with 60 patients at our clinic showed that patients’ acceptance of telemedicine during the first wave of the pandemic was high [7]. However, at the time, satisfaction was not recorded. As this is an important aspect of patient-centered care [8], satisfaction has become part of the core outcome domains for chronic pain clinical trials [9] and serves as an anchor for clinically important differences in pain intensity and other outcomes [10]. Further, high satisfaction is associated with the appropriateness of care [11] and contributes to therapy adherence [12]. Likewise, preferences were not assessed in our first investigation. However, as fulfillment of patients’ preferences is linked to better adherence to therapy and better allocation of resources [13], it is important to know what patients actually favor.

Furthermore, psychological factors have a big impact on pain-related outcomes: generalized anxiety disorders can be linked to poor global well-being and life satisfaction [14] and hypochondriac attitudes and beliefs have been shown to predict patients’ satisfaction [15]; therefore, these factors are important to take into account.

Finally, various studies provide evidence of the detrimental effect of physical distancing during the pandemic on mental health [16-19]. It is conceivable that governments’ restrictions influence patients’ acceptance and satisfaction with telemedicine.

This warranted a follow-up study to investigate (1) the levels of acceptance and satisfaction in the subsequent months, whether levels of acceptance changed after the first wave of the COVID-19 pandemic and what levels of satisfaction were achieved with telemedicine; (2) patients’ preferences for care during the pandemic and whether preferences differ in patients who had already experienced telemedicine; (3) correlations between acceptance and satisfaction, current pain intensity, general condition, as well as several measures of pain-related self-reports (anxiety, hypochondriacal worries, and catastrophizing) to assess whether these are associated with acceptance itself and patients’ preferences; and finally (4) correlation of acceptance with the severity of restrictive measures ordered by the government, to investigate whether more restrictions relate to higher acceptance.

## Methods

### Sample

We conducted an anonymous, voluntary survey between January 27, 2021, and February 4, 2022, at the Pain Center of the Bern University Hospital, Switzerland. Patients who were seen in person in our clinic received an invitation to complete the digital survey. Inclusion criteria were aged at least 18 years, with chronic pain, all conditions included. Patients with an acute illness requiring emergency procedures [20] were excluded.

### Procedure and Study Design

A cross-sectional web-based survey was created to assess patients’ satisfaction with telemedicine consisting of phone calls (for the full questionnaire see Table S1 in Multimedia Appendix 1). In contrast to our previous study [7], the current investigation was conducted after the shutdown and with patients who were seen in person during routine outpatient treatment at our Pain Center. Whereas our previous study only accounted for first contacts with patients over the phone, the follow-up survey also involved patients who had already received a phone consultation. These respondents additionally rated their satisfaction as well as their perceptions of various aspects of telemedicine. A questionnaire was placed at the reception of our outpatient pain service, and newly referred patients were encouraged to complete it by scanning a QR code. We conducted this study solely by relying on departmental resources, without external funding.

### Ethical Considerations

All the included patients provided informed consent for the reuse of their data. A jurisdictional inquiry was submitted to the Ethics Committee of the Canton of Bern, Switzerland (BASEC Req-2020-01406). The Ethics Committee decided that this study had no jurisdiction and was therefore exempted from ethics approval. Due to the anonymous assessment via QR code, it was impossible to identify individual patients. The participants received no financial compensation.

### Materials

First, participants provided demographic data (sex and age) and reported the intensity and duration of their pain, their general condition, and previous interventional pain treatments.

Subsequently, the perceived acceptance of telemedicine and other aspects of patients’ expectations concerning the COVID-19 pandemic were assessed on a Numeric Rating Scale (0=not at all and 10=completely), that is, regarding their confidence in dealing with pain and with the pandemic, whether they feared a severe COVID-19 infection, and whether they thought the health care system and politics had taken the correct steps to cope with the consequences of the pandemic.

The validated German version of the generalized anxiety disorder 2-item [21,22] was used to assess the level of general anxiety with 2 items (eg, “nervousness, anxiety, or tension”) on a Likert scale from 0 to 3, ranging from “not at all” (0) to “nearly every day” (3), leading to a total score of 0 to 6, with a cutoff value of 3 for a generalized anxiety disorder.

To measure pain catastrophizing, the German short form of the Pain Catastrophizing Scale (PCS-6) was used [23]. The PCS-6 consists of 6 items (eg, “I keep thinking about how much it hurts”) that are rated on 5-point Likert scales, ranging from “not at all” (0) to “all the time” (4), leading to a total score of 0-24.

The Whiteley Index [24] was used to assess hypochondriacal worries and beliefs using 6 items (eg, “Do you often worry about the possibility that you have got a serious illness?”), rated on a 5-point Likert scale, ranging from “not at all” (1) to “very much” (5), leading to a total score of 6-30 [24,25].

Respondents were then asked to choose their preferred type of consultation during the COVID-19 pandemic: in person, by telephone, or none. If they had already received a phone consultation, more questions were asked regarding satisfaction with and perception of telemedicine. Most of these items were evaluated on an 11-point Numeric Rating Scale from 0 (not at all) to 10 (completely), with the exceptions of the items “average pain intensity over the last 24 hours” (0=no pain and 10=worst pain imaginable) and “general condition” (0=very poor and 10=excellent).

As an evaluation of the strictness of government measures, the Oxford COVID-19 Government Response Tracker (the COVID-19 stringency index) was used [26]. This index uses 9 metrics to calculate the strictness of government policies: closures of public transport; closures of workplaces; restrictions on public gatherings; cancelation of public events; school closures; requirements to stay at home or to observe curfews; public information campaigns; restrictions on movements within the country; and international travel controls. Then, the strictness of government policies in each area was calculated on a scale from 0 to 100, with 100 being the strictest response. Subsequently, the mean score of the 9 metrics was calculated.

### Statistical Analysis

Our data were analyzed using descriptive and correlational statistics (absolute and relative frequencies, mean and SD, and median and IQR), primarily to assess acceptance of telemedicine. To check that our sample was representative, we compared the demographic data of our participants (sex, age, and pain intensity) with those of all patients who had been referred to the clinic during the study period. To analyze correlations with satisfaction, pain levels, general conditions, and other self-reported measures, as well as the severity of restrictive measures, a Spearman correlation was computed, with correlations of 0.2-0.4 considered weak, 0.4-0.6 moderate, and 0.6-0.8 strong [27].

Regarding preferences, we first analyzed them descriptively and then assessed whether they differed in participants who had previously received phone consultations, as well as whether there were differences between patients who had been treated for longer than three months at our clinic and newly treated participants. Using a chi-square test for categorical data, these groups were further evaluated with Cramer *V* statistic to assess effect sizes. A magnitude of 0.1 was considered small, 0.3 medium, and 0.5 large [28]. For continuous data, a 2-sided Student *t* test with equal variance or Welch *t* test in the case of unequal variance as indicated by the Levene test at *P*<.05 was calculated.

Descriptive analyses were performed with *jamovi* (version 2.2; jamovi project) [29]. Visualizations were performed using GraphPad Prism (version 8.0.0 for Windows, GraphPad Software), and RStudio Team (Posit). Correlations and corresponding graphs were computed in RStudio: Integrated Development for R (RStudio, PBC). Statistical significance was set at a *P* value of <.05.

## Results

### Demographic Data

The survey was accessible between January 27, 2021, and February 4, 2022. During this time, a total of 816 new patients were seen at our center. In total, 77 (9.4%) patients logged in, 60 (77.9%) patients of whom completed the questionnaire. Most participants were female (n=39, 65%) with an average age of 50.8 (SD 18.3) years (Table 1). About half of them (n=29, 48%) had been newly treated at the Pain Center, and the majority had already received phone consultations as part of their routine treatment (n=42, 70%). More than half of the respondents had previously received interventional pain therapy (n=31, 52%); however most procedures were either unsuccessful or had only provided short-term pain relief (n=24, 77%).

The mean pain intensity score was 6.3 (SD 2.3) and the general condition was reported to be 6.5 (SD 2.2; Table 2). A comparison with all patients who had been referred to the clinic (with acute and chronic conditions) during this study period revealed no statistically significant differences in terms of sex or age, indicating that our sample may be considered representative of all patients seen during this time (Table S2 in Multimedia Appendix 1).

**Table 1:** Baseline characteristics of all enrolled patients in 2021-2022.

Characteristics		Mean (SD)	n (%)
Age (years)		50.8 (18.3)	N/A^a^
Sex, female		N/A	39 (65)
Pain duration (years)		2.7 (0.6)	N/A
**Pain duration**			
	Below 3 months	N/A	3 (5)
	3 m to 1 years	N/A	13 (22)
	Over 1 years	N/A	44 (73)
**Interventional pain therapy**			
	No	N/A	25 (42)
	Yes, without success	N/A	13 (22)
	Yes, successful over the short term	N/A	11 (18)
	Yes, successful over the long term	N/A	7 (12)
	Not specified	N/A	4 (7)
Phone consultation received (yes)		N/A	42 (70)
**Treatment**			
	Newly treated	N/A	29 (48)
	Longer than 3 months	N/A	31 (52)

^a^N/A: not applicable.

**Table 2:** Descriptive analysis of patients’ responses.

Descriptive			Value
Pain^a^, mean (SD)			6.3 (2.3)
General condition, mean (SD)			6.5 (2.2)
Accept^b^, mean (SD)			7.6 (3.3)
PhonCov^c^, mean (SD)			7.7 (3.2)
Satis^d,e^, mean (SD)			8.8 (1.8)
PercSinc^d,f^, mean (SD)			9.1 (1.6)
Quest^g^, mean (SD)			9.2 (1.3)
HelpCons^d,h^, mean (SD)			7.9 (2.7)
Long-term improvement of pain, mean (SD)			6.8 (2.5)
Confidence in dealing with pain, mean (SD)			7.5 (2.2)
Confidence regarding COVID-19 pandemic, mean (SD)			9.0 (1.8)
Correct medical steps, mean (SD)			8.7 (1.9)
Correct political steps, mean (SD)			7.6 (2.4)
Adequate treatment of pain, mean (SD)			3.3 (3.4)
Fear of severe coronavirus infection, mean (SD)			4.0 (3.7)
**Preference, n (%)**			
	Preferably a consultation on-site	35 (58)	
	Preferably a phone consultation	24 (40)	
	No consultation	1 (2)	
Anxiety (GAD-2^i^, 0-6 points; n=17, 28%), mean (SD)			2.0 (1.7)
Pain catastrophizing (PCS-6^j^, 0-24 points; PCS6), mean (SD)			10.3 (6.1)
Frequent worries (WI-6^k^, 6-30 points; WI6), mean (SD)			7.0 (5.6)
Strictness of government response (scale 0-100), median (IQR)			51 (48-56)

^a^Pain: Average pain intensity.

^b^Accept: Acceptance of telemedicine.

^c^PhonCov: Phone consultation without COVID-19.

^d^Only calculated if patients had already received phone consultations.

^e^Satis: Satisfaction with telemedicine.

^f^PercSinc: Perception of sincerity.

^g^Quest: Questions were addressed.

^h^HelpCons: Could be helped by phone consultation.

^i^GAD-2: generalized anxiety disorder 2-item.

^j^PCS-6: Pain Catastrophizing Scale 6-item.

^k^WI-6: Whiteley Index 6-item.

### Acceptance and Satisfaction With Telemedicine

**Figure 1:**
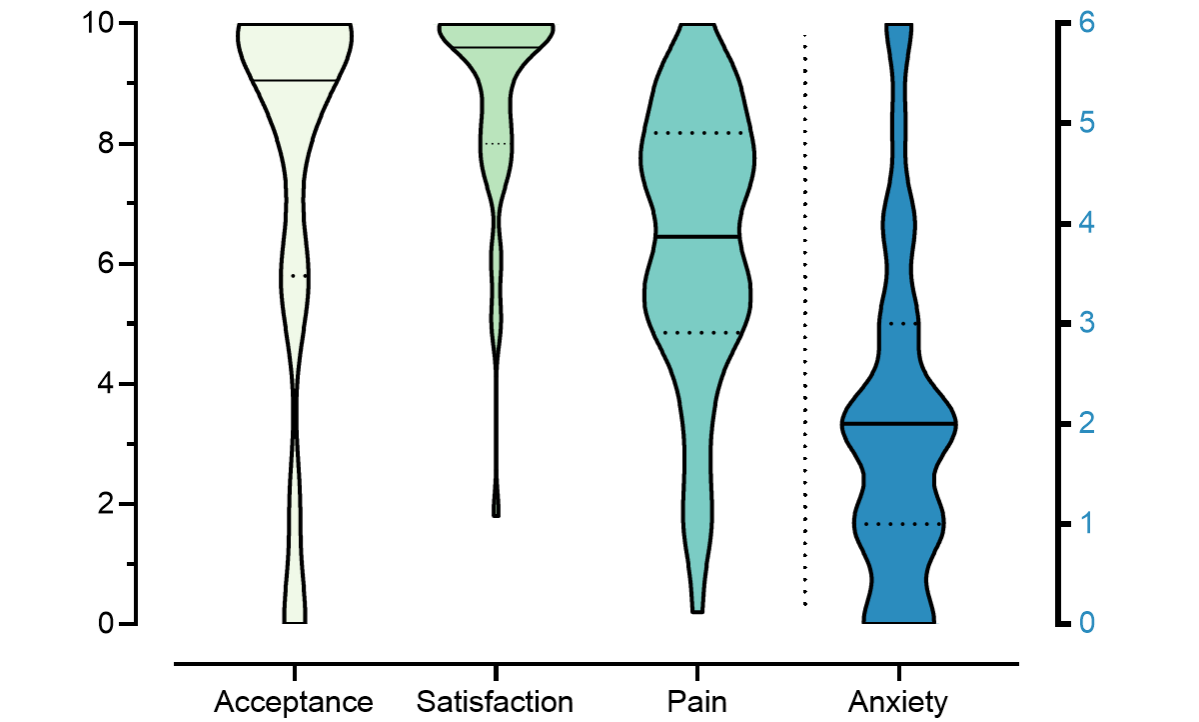
Baseline characteristics of satisfaction, acceptance, pain, and anxiety. Violin plots showing the distribution of respondents’ answers (N=60) on an 11-point scale for acceptance, satisfaction, and pain (left y-axis). On the right and separated, anxiety scores are indicated on the 6-point GAD-scale (right y-axis). Continuous line indicates the median and dotted lines the 1st and 3rd quartile. GAD: generalized anxiety disorder.

The acceptance of telemedicine varied among patients, with a mean score of 7.6 (SD 3.3). Most reported that they would also have made use of telemedicine without the necessity imposed by the COVID-19 pandemic restrictions (item “phone consultation without COVID-19,” mean 7.7, SD 3.2) and those patients who had received phone consultation reported high levels of satisfaction (mean 8.8, SD 1.8). These respondents felt that their concerns had been taken seriously (mean 9.1, SD 1.6), that their questions had been addressed (mean 9.2, SD 1.3), and that they could be helped (mean 7.9, SD 2.7). The distributions of acceptance, satisfaction, pain, and anxiety are displayed in Figure 1.

### Affective State and Processing of Pain During the COVID-19 Pandemic

Participants tended to think their pain would improve over the long term (mean 6.8, SD 2.5). They were even more optimistic concerning their ability to cope with pain (mean 7.5, SD 2.2) and with the COVID-19 pandemic (mean 9.0, SD 1.8). There was high variability in responses to the “fear of pain not being addressed adequately in the future” and “fear of a severe COVID-19 infection.”

Screening for anxiety using the generalized anxiety disorder 2-item showed low scores (mean 2.0, SD 1.7; see Table 2 and Figure 1). However, using a cutoff of ≥3 points [22], we found about 1 quarter (n=17, 28%) of our patients had a high likelihood of having generalized anxiety or panic disorder. Catastrophizing scores using the PCS-6 were generally high (mean 10.3, SD 6.1), while the assessment of hypochondriacal worries and beliefs (operationalized with Whiteley Index 6-item) showed rather low values (mean 7.0, SD 5.6).

### Patients’ Preferences for Care

In total, 24 participants (n=24, 40%) preferred telemedicine to on-site consultations (see Table 2). Nonetheless, a more detailed analysis revealed substantial differences between subgroups. The predilection for on-site consultation was distinctly higher in patients who had never had a phone consultation (14 of 18 patients, 78%), whereas half of the respondents who had already experienced telemedicine preferred on-site consultation over phone consultations (21 of 42 patients, 50%). A chi-square test showed a significant association with a medium effect size (*χ*^2^_2_ [N=60]=7.5, *P*=.02, Cramer *V*=0.354).

Likewise, participants who had been treated for less than three months tended to prefer on-site consultations (21 of 29 patients, 72%), whereas those treated for longer durations slightly preferred telemedicine over on-site consultations (17 of 31 patients, 55%). Here, the chi-square test was also significant with a medium effect size (*χ*^2^_2_ [N=60]=6.5, *P*=.04, Cramer *V*=0.329).

### Correlation Analysis

**Figure 2:**
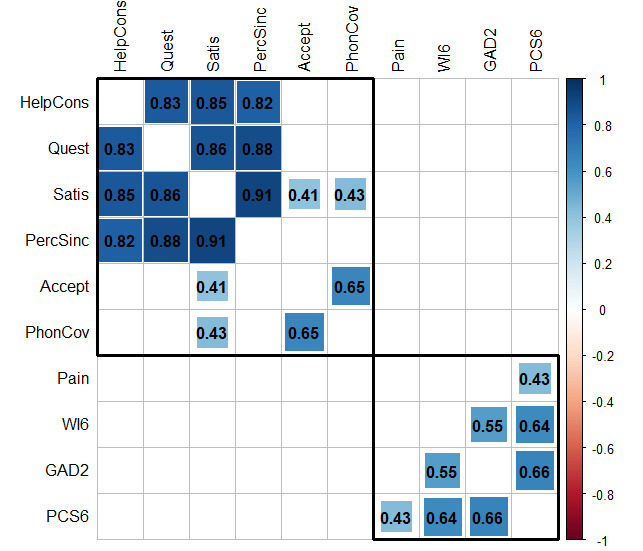
Correlation matrix of patients’ perceptions. Correlation plot for the associations between variables with a moderate to strong correlation (|*r*_s_|≥0.4). The colors and sizes of the boxes show the degree of pairwise correlation regarding Spearman rank correlation coefficient (*r*_s_). The results are clustered using the hierarchical clustering order of the "corrplot" R package (R Foundation for Statistical Computing). Accept: acceptance of telemedicine; GAD-2: generalized anxiety disorder 2-item; HelpCons: could be helped with phone consultation; Pain: average pain intensity; PCS6: Pain Catastrophizing Scale 6-item; PercSinc: perception of sincerity; PhoneConv: phone consultation without COVID-19; Quest: questions were addressed; Satis: satisfaction with telemedicine; WI6: Whiteley Index 6-item.

Acceptance of telemedicine showed a moderate positive correlation with satisfaction (*r*_s_{58}=0.41, *P*<.05) and a strong correlation with patients’ intention to use phone consultation outside of the COVID-19 pandemic as well (*r*_s_{58}=0.65, *P*<.05). In addition, in patients who had already experienced telemedicine, strong correlations were observed between satisfaction and quality-related items, such as the perception of sincerity and patients’ opportunities to clarify their questions (*r*_s_{58}=0.85-0.91, *P*<.05). Pain and psychometric scores showed moderate to high correlations. Figure 2 provides an overview of statistically significant results (*P*<.05) of the correlation analyses with at least a moderate strength (*r*_s_{58}≥0.4). There were no statistically significant correlations below moderate strength.

### Correlation of Acceptance With the Severity of Restrictive Measures

The COVID-19 stringency index reached a median of 50.93 (IQR 48.15-56), and measures remained largely the same over the course of this study period. The severity of restrictive measures did not correlate significantly with acceptance of telemedicine or any other investigated items (satisfaction, pain, general condition, catastrophizing, anxiety, hypochondriacal worries and beliefs, confidence regarding the COVID-19 pandemic, fear of severe coronavirus infection, or impressions regarding correct medical or political steps).

## Discussion

### Principal Findings

This study sheds light on the clinical reality of the COVID-19 pandemic, including how remote services are perceived and what patients actually prefer. We found high acceptance and satisfaction levels of telemedicine. Nevertheless, respondents generally favored on-site consultations, while our subgroup analysis revealed that this was associated with whether they had already received a phone consultation or had been treated for more than three months at our Pain Center. Acceptance was moderately associated with satisfaction and strongly associated with willingness to use telemedicine outside of the COVID-19 pandemic. None of the assessed items were associated with the strictness of measures against the COVID-19 pandemic. Compared to our previous investigation [7], the general condition was rated slightly higher in our sample, while the average pain intensity was comparable.

### Acceptance, Satisfaction, and Preferences

Our findings underscore a central point: acceptance and satisfaction are not the same as preference. This seems to be overlooked often, as most studies have not assessed what patients favor when offered several options for consultation. Some might accept and be satisfied with 1 service but still prefer the other [30]. For example, a recent investigation found high acceptability of a telemedicine pilot initiative for patients with chronic noncancer pain, but patients’ preferences were not examined specifically [31]. It is important to note that in our study, patients who had never experienced telemedicine or had only been treated for several weeks, preferred on-site consultations. One potential explanation could be that telemedicine is still largely unknown or underused by many patients. This is in line with a previous study that showed that one of the main reasons for not using telehealth was a lack of knowledge about it [32]. The good health coverage in Switzerland may contribute to this factor, as it is easier to find a timely appointment in person than in countries with more remote areas. Another reason could be barriers to accessing telemedicine in the senior population [33], who may not be as familiar with modern or unconventional modes of care. An additional factor could be trust in the practitioner, which has been shown to improve satisfaction as well as other patient-reported outcomes, such as quality of life [34]. In our case, it is conceivable that patients who had already had a telephone consultation and had a good experience gained more trust. The same applies to people who have been in treatment for longer than 3 months; they have had more time to gain trust in the treating doctor.

In summary, telemedicine acceptance in our study was high and comparable to other investigations that have examined acceptance in other collectives: for example, 1 study in patients who were urogynecologic found 87% acceptance [35], while 2 scoping reviews found high acceptance of telemedicine in palliative care [36] and older adult patients with tumors [37]. In another cross-sectional study of patients with tumors, high satisfaction was found, with a mean of 5.5 out of 7 [38]. Nevertheless, while acceptance of and satisfaction with telemedicine were high in these investigations, these findings cannot be applied to every patient as a “one-size-fits-all” rule, as patients’ individual preferences (and needs) might differ. This was exemplified in a recently published systematic review with meta-analysis on outcomes of web-based pain management for chronic widespread musculoskeletal conditions where the investigators found clinically significant improvements but failed to show a clinically relevant change [39]. Based on our findings, we would recommend asking patients specifically what service and mode of treatment they prefer and to build upon personal therapeutic relationships, thus improving patient-centered care.

Furthermore, the COVID-19 pandemic has led to barriers to seeking emergency care [40], and this should be considered as promoting the implementation of digital services, such as telemedicine and digital triage [41]. Nevertheless, it is currently an open question how telemedicine will continue to develop in the Swiss population after the first waves of the COVID-19 pandemic, and to what extent the initiated changes will last. Research on the future use of telehealth, which considers the experience of COVID-19 and the impact of the COVID-19 pandemic on health care providers and patients, is essential.

### Correlation Analyses

The values regarding perception of care were highly correlated with each other and satisfaction. While this is an unsurprising result, it highlights the importance of maintaining high-quality services, directly translating into how satisfied patients feel. Concerning the COVID-19 stringency index, we were unable to show any meaningful influence on satisfaction, acceptance, preferences, or the perception of treatment, which does not support the idea that patients are substantially driven by their perception of the “strictness” of governmental measures. Previously, we hypothesized that due to government restrictions, acceptance of and preference for telemedicine would be higher (as it is a measure of reducing personal contacts). Another explanation for the lack of correlation could be that during this study period, government measures were largely stable and varied only mildly, whereas, in theory, they could range between scores of 0 and 100. Therefore, the stringency index may show associations as soon as it exhibits greater change.

### Limitations

Our study is limited by the small sample size and the low response rate, which is a problem for reliability and generalizability and can lead to a nonresponse bias. We attempted to address this by comparing the general patient population seen at our clinic with our study respondents to ensure representativeness. One reason why so few patients participated could be the number of standard questionnaires that they received before an appointment at our clinic. It is conceivable that this could lead to reluctance to fill out additional forms if they are not necessary for treatment. Additionally, as this was a web-based survey only, this could exclude patients who are not familiar with QR code scanning and handling of technical devices [42]. It is also important to note that measuring acceptance and satisfaction on an 11-point scale only allows a very general impression with no further information on why telemedicine was accepted or rejected. By contrast, validated questionnaires [43,44] could paint a more accurate picture. This limitation was somewhat mitigated by the fact that we also asked whether the patients felt taken seriously, whether their questions were answered, whether they could be helped, and whether they would use telemedicine even without the COVID-19 pandemic. These items were all strongly correlated with satisfaction, which can be interpreted as an indication of their importance to satisfaction itself. Additionally, this study lacked information on socioeconomic status, income, level of education, digital competence, and literacy. This is important to consider in the overall context, as lower literacy, for example, can be seen as a significant factor in the failure of a medical intervention. Future studies should consider these points to evaluate a more comprehensive assessment of access to health care, including telemedicine. A possible template for such a study is the Levesque access framework, which, in addition to the determinants of health care (approachability, acceptability, availability or accommodation, affordability, and appropriateness), also takes into account the individual possibilities of patients and can thus capture possible barriers to health care in a differentiated way [45,46]. The qualitative or quantitative assessment of these aspects is likely to play an increasingly important role in the future, especially in the implementation of more modern technologies, such as chatbots [47] or other automated systems controlled by artificial intelligence [48]. In our view, the evaluation of patient acceptance and satisfaction in these areas is crucial for ensuring patient-centered care. In patients with chronic pain, it would be interesting to investigate whether there are patient groups that particularly benefit from telemedicine (for example, those with limited mobility, more comorbidities, those with frequent medication adjustments, or the need for a higher frequency of care, such as cancer pain). Furthermore, the term “telemedicine” is widely used nowadays, and in our case, it was applied only to phone consultations. It is possible that participants in other outpatient settings using other services such as video chats might experience telemedicine differently and prefer these methods. Owing to administrative and legal hurdles in Switzerland, the general introduction of video consultations remains a challenge in the near future. Finally, options for billing patients are restricted to a maximum time limit of 20 minutes for an appointment by phone or video call according to the Swiss tariff structure [49].

### Conclusions

Although telemedicine was widely accepted in our patient population and patients were generally very satisfied with it, they nevertheless preferred on-site consultations. In contrast, patients who had already experienced telephone consultation or those who had already undergone treatment for more than three months showed significantly higher preferences for telemedicine. This highlights 2 points: first, knowledge of eHealth services needs to be conveyed to patients to make use of its many advantages. Second, our investigation stresses the importance of building meaningful relationships with patients at the beginning of treatment and how this can improve patients’ perception of care. During the COVID-19 pandemic, the modality of care should be discussed individually for each patient.

## Appendix

Multimedia Appendix 1 Table S1 and Table S2.

## Abbreviations

PCS-6: Pain Catastrophizing Scale 6-item

## Data Availability

The data used to support the findings of this study are restricted by the Cantonal Ethics Committee of Bern, Switzerland, to protect patient privacy. Data are available upon reasonable request from MAH, Doctor of Medicine, for researchers who meet the criteria for access to confidential data.
