# Supplementary material for: Acceptance, Satisfaction, and Preference With Telemedicine During the COVID-19 Pandemic in 2021-2022: Survey Among Patients With Chronic Pain
Source: JMIR Form Res. 2024 Apr 29;8:e53154. doi: 10.2196/53154 (PMC11060324; doi:10.2196/53154)
Supplement: Multimedia Appendix 1 [file formative_v8i1e53154_app1.docx]

**Table S1.** Questionnaire.

| **General condition** |
| --- |
| My current general condition is… (0 = very poor, 10 = excellent) |
| **Average pain intensity [Pain]** |
| Over the last 24 hours my pain intensity was on average… (0 =no pain, 10 = worst pain imaginable) |
| **Acceptance of telemedicine [Accept]** |
| I consider telephone consultations to be feasible and appropriate for me in the current situation. (0 = not at all, 10 = completely) |
| **Phone consultation without COVID [PhonCov]** |
| I would use phone consultation even without the Corona pandemic. (0 = not at all, 10 = completely) |
| **Satisfaction [Satis]*** |
| Overall, I’m satisfied with the phone consultations. (0 = not at all, 10 = completely) |
| **Perception of sincerity [PercSinc]*** |
| I felt that my concerns were taken seriously during the phone consultation. (0 = not at all, 10 = completely) |
| **Questions were addressed [Quest]*** |
| During the phone consultation, all of my questions were addressed. (0 = not at all, 10 = completely) |
| **Could be helped with phone consultation [HelpCons]*** |
| I was sufficiently helped with the phone consultation. (0 = not at all, 10 = completely) |
| **Long-term improvement of pain** |
| I think my pain will improve over the long term. (0 = not at all, 10 = completely) |
| **Confidence in dealing with pain** |
| I am confident that I will cope with my pain. (0 = not at all, 10 = completely) |
| **Confidence regarding Corona Pandemic** |
| I am confident that I will overcome the Corona Pandemic with all its consequences. (0 = not at all, 10 = completely) |
| **Correct medical steps** |
| I have the general impression that the healthcare sector has taken the right steps so far during the pandemic. (0 = not at all, 10 = completely) |
| **Correct political steps** |
| I have the impression that our political leaders have taken the right steps so far during the pandemic. (0 = not at all, 10 = completely) |
| **Anxiety regarding inadequate treatment of pain during Corona crisis** |
| I am afraid that my pain will not be adequately treated during the Corona crisis. (0 = not at all, 10 = completely) |
| **Fear of severe coronavirus infection** |
| I’m afraid of becoming seriously ill due to COVID-19. (0 = not at all, 10 = completely) |
| **Preference** |
| In times of the Corona pandemic, I prefer: (1= consultation on-site, 2= phone consultation, 3= no consultation) |
| **Improvement with a phone consultation** |
| Could something about the phone consultation be improved? If so, what (open format) |
| **Generalized Anxiety Disorder Item 1** |
| Over the last 2 weeks, how often have you been bothered by feeling nervous, anxious or on edge (0= not at all, 1= several days, 2= more than half the days, 3= nearly every day) |
| **Generalized Anxiety Disorder Item 2** |
| Over the last 2 weeks, how often have you been bothered by not being able to stop or control worrying (0= not at all, 1= several days, 2= more than half the days, 3= nearly every day) |
| **Pain Catastrophizing Item 1** |
| It`s awful and I’m overwhelmed (0= not at all, 1= to a slight degree, 2= to a moderate degree, 3=to a great degree, 4= all the time) |
| **Pain Catastrophizing Item 2** |
| I feel I can`t stand it anymore (0= not at all, 1= to a slight degree, 2= to a moderate degree, 3=to a great degree, 4= all the time) |
| **Pain Catastrophizing Item 3** |
| I become afraid that the pain may get worse (0= not at all, 1= to a slight degree, 2= to a moderate degree, 3=to a great degree, 4= all the time) |
| **Pain Catastrophizing Item 4** |
| I keep thinking about how much it hurts (0= not at all, 1= to a slight degree, 2= to a moderate degree, 3=to a great degree, 4= all the time) |
| **Pain Catastrophizing Item 5** |
| I keep thinking about how badly I want the pain to stop (0= not at all, 1= to a slight degree, 2= to a moderate degree, 3=to a great degree, 4= all the time) |
| **Pain Catastrophizing Item 6** |
| I wonder whether something serious may happen (0= not at all, 1= to a slight degree, 2= to a moderate degree, 3=to a great degree, 4= all the time) |
| **Whiteley Index Item 1** |
| Do you often worry about the possibility that you have a serious illness (1= not at all, 2= to some extent, 3= moderately, 4=to a considerable extent, 5= to a great extent) |
| **Whiteley Index Item 2** |
| Do you worry a lot about your health (1= not at all, 2= to some extent, 3= moderately, 4=to a considerable extent, 5= to a great extent) |
| **Whiteley Index Item 3** |
| If a disease is brought to your attention (e.g. on TV, radio, the newspapers, or by someone you know), do you worry about getting it yourself (1= not at all, 2= to some extent, 3= moderately, 4=to a considerable extent, 5= to a great extent) |
| **Whiteley Index Item 4** |
| Do you think that you worry about your health more than most people (1= not at all, 2= to some extent, 3= moderately, 4=to a considerable extent, 5= to a great extent) |
| **Whiteley Index Item 5** |
| Do you think there is something seriously wrong with your body (1= not at all, 2= to some extent, 3= moderately, 4=to a considerable extent, 5= to a great extent) |
| **Whiteley Index Item 6** |
| Are you afraid of illness (1= not at all, 2= to some extent, 3= moderately, 4=to a considerable extent, 5= to a great extent) |

*only presented if patients already had already received a phone consultation.

**Table S2.** Demographics of study respondents and general population.

|  | **Questionnaire** (N=60) | **General** (N=816) | ***P*-value** |
| --- | --- | --- | --- |
| Female | 39 (65.0) | 495 (60.7) | .51 |
| Male | 21 (35.0) | 321 (39.3) | .51 |
| Age in years | 51±18 | 50±20 | .55 |

Categorical data are shown as n (%), continuous data are calculated as mean±SD. A *P*-value of less than .05 was considered significant.
